# Cooperativity leads to temporally-correlated fluctuations in the bacteriophage lambda genetic switch

**DOI:** 10.3389/fpls.2015.00214

**Published:** 2015-04-08

**Authors:** Jacob Q. Shenker, Milo M. Lin

**Affiliations:** ^1^Department of Physics, California Institute of TechnologyPasadena, CA, USA; ^2^Pitzer Center for Theoretical Chemistry, University of CaliforniaBerkeley, CA, USA

**Keywords:** gene regulatory networks, markov state models, phage lambda, mutual information, information theory, conditional activity

## Abstract

Cooperative interactions are widespread in biochemical networks, providing the nonlinear response that underlies behavior such as ultrasensitivity and robust switching. We introduce a temporal correlation function—the *conditional activity*—to study the behavior of these phenomena. Applying it to the bistable genetic switch in bacteriophage lambda, we find that cooperative binding between binding sites on the prophage DNA lead to non-Markovian behavior, as quantified by the conditional activity. Previously, the conditional activity has been used to predict allosteric pathways in proteins; here, we show that it identifies the rare unbinding events which underlie induction from lysogeny to lysis.

## 1. Introduction

Cells use biochemical networks to sense, process information, and respond to their environments. Many cellular behaviors have been found to be controlled by genetic switches, in which the expression levels of a set of genes form a stable memory of a transient stimulus, allowing the cell to make a decision and remember it. These networks range from a simple bistable switch to complicated networks involving dozens of genes and many stable states of the switch (fixed points). In bacteria and viruses, these switches enable phenotypic switching to optimize the fitness of the organism in response to environmental conditions. In temperate bacteriophages, a switch selects between dormancy and virulence, and in *Escherichia coli*, bistable switches are known to regulate the lactose (Ozbudak et al., 2004) and arabinose utilization systems (Fritz et al., 2014). More complex gene regulatory networks are used in plants and animals to coordinate development and determine cell fates. In *Arabidopsis thaliana*, a fifteen-gene network was identified whose fixed points correspond to the ten flower cell types (Espinosa-Soto et al., 2004). This is typical of many gene regulatory networks, where the phenotype corresponds not to expression of any single gene, but rather to the collective state of the system. These systems are challenging to study theoretically and experimentally, since the effects of a given gene on phenotype is difficult to disentangle from the behavior of the other genes in the network. We propose a theoretical technique which identifies the functional connectivity between different elements in a biochemical network. This map elucidates the often non-intuitive connection between genotype and phenotype in these networks, and may be used to design experimental interventions which most effectively modify or disrupt this collective behavior, and hence most directly affect phenotype.

The multistability which characterizes these switch-like gene networks often results from the interplay of feedback and ultrasensitivity. These dynamics arise from individual binding interactions between the nucleic acids, proteins, and small ligands that comprise these networks. Information propagates between these species through macromolecular complexes containing multiple binding sites. The presence of a ligand at one binding site modulates the activity or binding strength of another binding site through cooperativity or conformational change (allostery). Allosteric regulation has been widely and successfully described by the Monod-Wyman-Changeux (MWC) model (Monod et al., 1963), while cooperativity and other forms of indirect regulation have been described by generalizations thereof (Marzen et al., 2013). These models describe the thermodynamic equilibrium of these systems. For the two-site MWC model, cooperative binding energies can be fit to experimental binding curves. However, the number of possible cooperative interactions increases rapidly with the number of binding sites, and for complex systems these interactions are often too numerous to constrain experimentally. In the cases for which it is not possible to fit a model, thermodynamic correlations within the system can still be inferred by observing the system and calculating the mutual information between its binding sites. However, for systems stabilized by cooperative binding, important behavior may be invisible to thermodynamic correlations and evident only from the timing of binding events.

Inspired by the physics of glasses, we introduce a new quantity, the conditional activity, to measure temporal correlations of binding activity in a biochemical system. The conditional activity can be calculated from direct experimental measurements or from a stochastic model. Recently, we used the conditional activity to measure temporal correlations between different regions of proteins as calculated from molecular dynamics simulations (Lin, under review). For these proteins, the conditional activity correctly distinguished functional modules and identified the allosteric connections between sites within a protein. This type of intramolecular communication was not detectable using mutual information or other equilibrium-based correlation functions.

In this contribution, we go beyond the molecular scale and apply the conditional activity to study temporally-correlated binding activity in a gene regulatory network. In these networks, complex system-level behaviors—such as adaptation, switching, and oscillation—arise from macromolecular binding of transcription factors to DNA and the resulting modulation of gene transcription, and this binding is often subject to cooperativity and competition between transcription factors. We consider the genetic switch in *E. coli* infected with bacteriophage lambda, whose bistability emerges from cooperativity between six binding sites, including long-range DNA looping. The switch reliably maintains its initial pathway—lysogeny—until it is flipped into another pathway—lysis—by an external trigger (the bacterium's SOS response).

**Figure 1:**
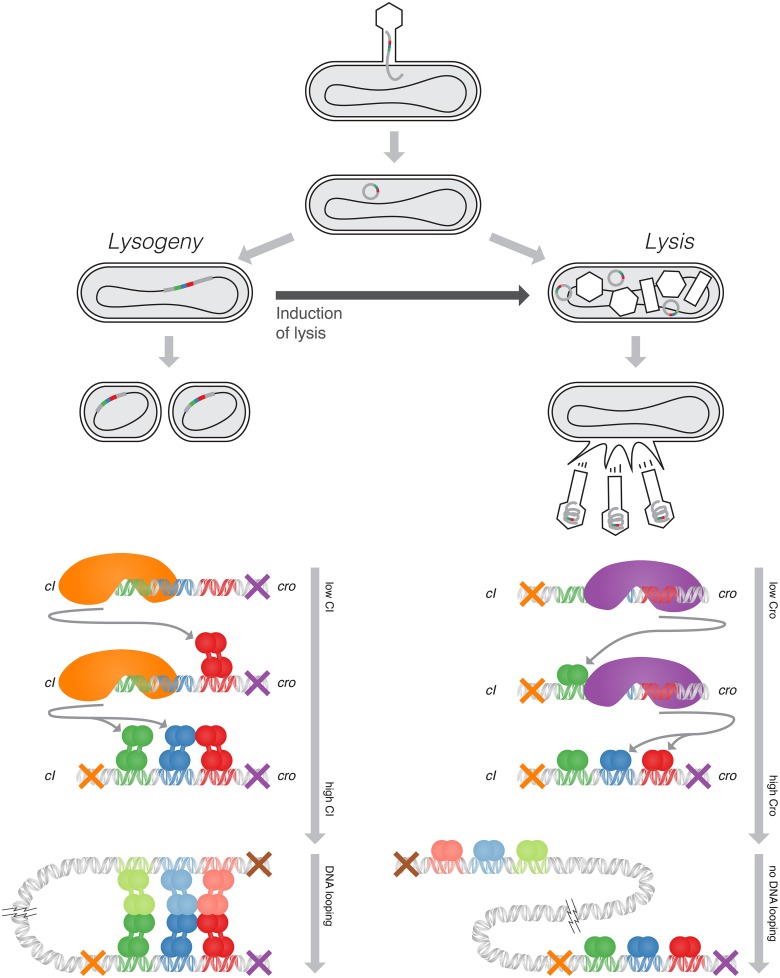
**Top**: The lifecycle of lambda phage, showing the lysis and lysogeny pathways. **Bottom, Left**: The negative-feedback loop active in the lysogenic pathway, maintaining a constant level of CI, and one of the looping configurations which exhibits at high CI concentrations. **Bottom, Right:** The negative-feedback loop active in the lysis pathway, maintaining a constant level of Cro. DNA does not loop under lytic conditions. (Adapted from Ptashne, 2004).

Lambda, a bacterial virus, infects an *E. coli* cell, and depending on the environment inside the *E. coli* proceeds along one of two pathways (Figure 1 top). In the more-common lytic pathway, the virus DNA enters free-floating into the bacterium. The host bacterium's own machinery replicates and synthesizes protein from the viral DNA, which in turn self-assemble into new bacteriophages. When approximately one hundred progeny phage have been produced, the viral DNA produces proteins that rupture the bacterial cell and the newly-made phages are released into the environment. In some cases, however, the viral DNA integrates itself into the host DNA and lies dormant—this is the lysogenic pathway. Once there, it is replicated along with the host DNA and is passed on to daughter cells upon division. The dormant state is exceedingly stable, and the virus may lie dormant for hundreds of millions of generations without activation (Little and Michalowski, 2010). However, the bacterium's SOS response, triggered by DNA damage, reliably induces the activation of the virus genes, and the virus switches over to the lysis pathway, replicating itself before it lyses its host.

Upon infecting its host bacterium, the lambda phage chooses one of these two pathways. This choice is maintained by a bistable genetic switch composed of the *cI* gene, the *cro* gene, and an operator that modulates the rate of their transcription. The CI and Cro proteins, upon dimerizing, become transcription factors which bind to this operator region. The lysogenic pathway is characterized by expression of *cI* and inactivation of *cro*, whereas the lytic pathway is initiated by expression of *cro*, which in turn inactivates *cI*. CI and Cro each negatively autoregulate, maintaining stable levels of protein when their gene is turned on (Figure 1 bottom). The cooperativity and competition between the binding of CI and Cro to the operator couples these negative-feedback loops, resulting in switch-like behavior.

We analyze this system looking for correlations in binding state between different binding sites, using the mutual information, and correlations in binding times (i.e., non-Markovian behavior), using the conditional activity. Since cooperative binding leads to stability, we find that the mutual information fails to describe any nontrivial relationships between binding sites because mutual information is small for stable systems. The conditional activity, however, is sensitive to rare collective fluctuations of the system and uses this information to infer a rich network of interactions between binding sites.

## 2. Materials and methods

### 2.1. Model

We adopt a standard model for the phage lambda switch (Ackers et al., 1982; Shea and Ackers, 1985; Santillán and Mackey, 2004; Tian and Burrage, 2004; Gedeon et al., 2008). The region of the lambda phage DNA relevant to this switch contains the right operator with three binding sites (*O*_R1_, *O*_R2_, *O*_R3_) sandwiched between two promoter regions (*P*_R_ and *P*_RM_, governing transcription of *cI* and *cro*, respectively), and the left operator with three binding sites (*O*_L1_, *O*_L2_, *O*_L3_) and a promoter region (*P*_L_, whose function is immaterial here). Each operator site can be bound by a CI dimer, a Cro dimer, or left unbound. Each promoter region may be bound by RNA polymerase (RNAP) or left unbound. The promoter regions physically overlap the operator: RNAP bound to *P*_R_ blocks anything from binding to *O*_R3_; RNAP bound to *P*_RM_ blocks binding at both *O*_R1_ and *O*_R2_; and RNAP bound to *P*_L_ block binding at *O*_L1_ and *O*_L2_. All possible binding configurations of these nine sites give 1200 microstates.

The energies of binding and cooperativity of CI, Cro, and RNAP have been measured experimentally (Reinitz and Vaisnys, 1990; Darling et al., 2000a,b), and from these we may calculate the free energies Δ*G*_*i*_ of each microstate *i*. The probabilities that the system will be found in a microstate *i* is given by

(1) $$K_{i}=\frac{1}{Z}\exp(-\Delta G_{i}/RT){\left[CI_{2}\right]}^{\alpha_{i}}{\left[Cro_{2}\right]}^{\beta_{i}}{\left[RNAP\right]}^{\gamma_{i}}$$

where α_*i*_, β_*i*_, γ_*i*_ are the numbers of bound CI, Cro, and RNAP in each microstate *i*, [*x*] indicates the concentration of *x*, *R* is the universal gas constant and *T* = 310 *K* is the temperature. The partition function *Z* is a normalization chosen to ensure that the system lies in one of the microstates at all times, i.e., that the probabilities sum to one: $\sum_{i=1}^{1200}K_{i}=1$.

As we show below, this model exhibits behavior under lysogenic conditions that is qualitatively different from that under lytic conditions (see Results). This difference arises because lysogeny and lysis occur at different concentrations of CI and Cro, yielding different equilibrium probabilities *K*_*i*_. The concentrations of CI, Cro, and RNAP that correspond to the lysogenic and lytic pathways are obtained using the model and parameters from Santillán and Mackey (2004) (see Supplementary Material for details and parameter values). We choose a repressor degredation rate γ_cI_ = 0.015 min^−1^, which lies in the range consistent with bistability.

Note that CI bound to *O*_R2_ increases the transcription rate of CI elevenfold when RNAP is bound to *P*_RM_. This cooperativity between CI and RNAP is a key interaction in phage lambda. However, our analysis encompasses only the behavior of the system on the fast timescale of macromolecular binding and unbinding, and not the far slower timescale of protein production. As such, in this work we consider cooperativity only in the sense of cooperative binding. Furthermore, because our model does not model the dynamics of protein production and degredation, our model cannot transition between lysogenic and lytic pathways by varying the protein concentrations, as the protein concentrations are held fixed at either lysogenic or lytic conditions. However, we examine the tendency for the system to switch from lysogeny to lysis by considering the binding of RNAP to *P*_R_. The more often *P*_R_ is bound by RNAP, the faster Cro is being produced, pushing the system toward lysis.

The probabilities *K*_*i*_ describe the occupancies of a system in thermodynamic equilibrium. For each of the two pathways, we now construct a continuous-time Markov chain which adds kinetic information—namely, the rates of transitions between microstates—to this thermodynamic model. In these Markov models, each transition corresponds to a single binding or unbinding event. We consider the substitution of one ligand for another as two distinct transitions: an unbinding of the first ligand followed by a binding of the second ligand. Since the binding and unbinding rates have not been directly determined experimentally, we infer approximate kinetics from the thermodynamics. Assuming detailed balance fixes the ratio between the forward (*q*_*ij*_) and backwards (*q*_*ji*_) rates for each transition, *q*_*ij*_/*q*_*ji*_ = *K*_*j*_/*K*_*i*_. We are free to choose the *q*_*ij*_ so that binding events have the same rate *D*:

(2) $$q_{ij}=\{\begin{array}{ll} D & \;i\to j\text{ is a binding transition}\\ D\frac{K_{j}}{K_{i}} & \;i\to j\text{ is an unbinding transition} \end{array}$$

Here we have assumed detailed balance and equal binding rates *D* to derive the kinetics *q*_*ij*_ from the equilibrium occupancies *K*_*i*_. This approximation is reasonable because all binding rates represent the physical process of diffusion-limited binding of macromolecules to DNA. Furthermore, not only was our choice of rates the simplest and most natural consistent with detailed balance, but we also have found that our results are highly insensitive to large deviations in these rates.

**Figure 2:**
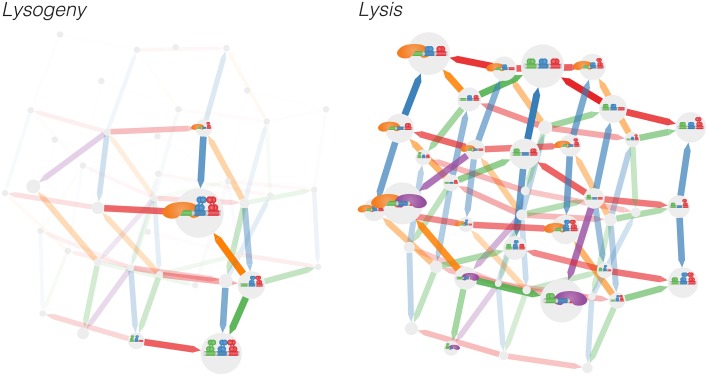
**The 1200 microstates of the left and right operator complex are projected down to the space of the 40 microstates of the right operator complex and the transitions between them for the (left) lysogeny and (right) lysis pathways**. Thus, the occupancies for each of the 40 microstates of the *right* operator complex shown is the sum of the occupancies of the 30 microstates that correspond to different microstates of the *left* operator complex. A binding event occurs in the direction of each arrow; unbiding occurs backwards along arrows. Arrows are colored according to the binding site experiencing a binding/unbinding event. The sizes of the microstates indicate the occupancy of the microstate, whereast the widths and transparency of the arrows represent the flux between microstates. Note that for the purpose of depicting the model, we have applied a highly nonlinear scaling; the four largest microstates account for the vast majority of occupancy (see Supplementary Figure 1).

In Figure 2 we show the Markov model by illustrating each microstate and the transitions between them, where the size of each microstate (gray circles) is proportional to the logarithm of its occupancy probability *K*_*i*_ and the width of each arrow is proportional to the logarithms of the flux (*K*_*i*_*q*_*ij*_) between two microstates. Even with this simple model of nine binding sites and three species of ligands, the resulting graph is complex and the functionality of the system is not evident: from the graph, it is difficult to discern how different binding sites couple to one another. To learn about the connections between binding sites, we may use a correlation function to project the connections between the 1200 microstates into the smaller space of nine binding sites. Note that the transitions between the 1200 microstates are Markovian; the non-Markovian dynamics arise when the model is projected into the space of the nine binding sites. In most experimental contexts it is not possible to observe all binding sites simultaneously, so the binding dynamics of the subset of binding sites which is observed appears non-Markovian. The conditional activity uses these non-Markovian dynamics to infer functional connectivity between the binding sites.

We now define two correlation functions with the aim of demonstrating that they capture orthogonal and complimentary information about a molecular system. In Section 2.2, we review the mutual information, a widely used correlation function which is useful for characterizing the dominant behavior of a system. We then define the conditional activity, which we introduced in Lin (under review), and is sensitive to fluctuations away from the dominant behavior. In Section 2.3, we demonstrate how the conditional activity may be directly calculated from the transition rate matrix for a Markov model of a molecular system; these correlation functions can also be calculated from simulating the Markov model, as described in Section 2.4.

### 2.2. Correlation functions

The mutual information is commonly used to infer connectivity in networks (Margolin et al., 2006). Here we use it to deduce the coupling between different binding sites. The mutual information (Shannon and Weaver, 1949; Cover and Thomas, 2006) between two binding sites *a*, *b* is defined as

(3) $$\begin{array}{l} MI(a,b)=\sum_{x\;\in \;X}P(a=x)\log_{2}P(a=x)\\ \;+\sum_{y\;\in \;Y}P(b=y)\log_{2}P(b=y)\\ \;-\sum_{x\;\in \;X}\sum_{y\;\in \;Y}P(a=x,b=y)\log_{2}P(a=x,b=y) \end{array}$$

where site *a* takes on binding configurations *X*, site *b* takes on binding configurations *Y*, and *P* is the probability function. Note that *P* can be written in terms of the *K*_*i*_: *P*(*a* = *x*, *b* = *y*) = $\sum_{i\;=\;1}^{1200}K_{i}\chi_{a,x}^{i}\chi_{b,y}^{i}$, where χ^*i*^_*a,x*_ is 1 if site *a* is in binding configuration *x* in system microstate *i* and 0 otherwise. We see that the mutual information is entirely a thermodynamic quantity, since it depends only on the equilibrium occupation probabilities *K*_*i*_.

The conditional activity is defined for systems with degrees-of-freedom that take on discrete configurations (or may be sensibly discretized), and captures correlations in the times between the times at which different degrees-of-freedom transition between configurations. As such, the CA is sensitive to the kinetics of the system, not only the thermodynamics. In this case, the degrees-of-freedom are the different binding sites, and each binding/unbinding event is considered a transition. Let *T*(*a*, *t*) be the *t*-th transition time of binding site *a* and τ ≡ *T*(*a*, *N*(*a*)) the duration of the observation period, with *N*(*i*) ≫ 1 being the number of recorded transitions. *W*(*a*, *T*) is the time between time *T* and the next transition of *a* after *T* [e.g., *W*(*a*, *T*(*a*, *t*)) = *T*(*a*, *t* + 1) − *T*(*a*, *t*), the time between a given binding event and the next unbinding event, or the time between a given unbinding event and the next binding event].

We define the mean observed persistence time to be half the mean squared waiting time between transitions for a given binding site *a*:

(4) $$\tau_{p}[a]\equiv \frac{1}{2\tau }\sum_{t\;=\;1}^{N(a)}W{\left(a,T(a,t)\right)}^{2}$$

For each transition time of binding site *b*, there is a previous transition of *b* and a next transition of *a*. These three times define two adjacent time periods, and the mean observed exchange time for *a* following *b* is:

(5) $$\tau_{x}[a\leftarrow b]\equiv \frac{1}{\tau }\sum_{t=1}^{N(b)-1}W(a,T(b,t+1))W(b,T(b,t))$$

The persistence time τ_*p*_[*a*] and exchange time τ_*x*_[*a* ← *a*] correspond to standard notions in glassy physics (Jung et al., 2005; Hedges et al., 2007); here we extend the exchange time to the case when *a* ≠ *b*. We now define the conditional activity of *a* following *b*:

(6) $$CA[a\leftarrow b]\equiv -\log_{10}\frac{\tau_{x}[a\leftarrow b]}{\tau_{p}[a]}$$

If *a* and *b* are independent, then *CA*[*a* ← *b*] = 0; if the transitions of *a* are Markovian, *CA*[*a* ← *a*] = 0. Note that the conditional activity, unlike the mutual information, is asymmetric: in general, *CA*[*a* ← *b*] ≠ *CA*[*b* ← *a*], because the conditional activity depends on the time-ordering of transitions.

For a real system, the occupancy probabilities *K*_*i*_ may be measured experimentally; for the Markov model, they are given in Equation (1). From these, the mutual information may be calculated using Equation (3). Similarly, using Equation (6), the conditional activity may be obtained from a time-series of configuration changes for each binding site. This time-series may be obtained experimentally for a real system. For the Markov model, we may generate this time-series data by simulating the continuous-time Markov chain using the Gillespie algorithm (Gillespie, 1977). However, the conditional activity is sensitive to rare events, so a long simulation is required to accurately calculate the conditional activity. For our simple Markov model, this simulation is still very tractable, but would pose a problem for more complicated models. We now use techniques of Markov chain theory to express the conditional activity directly in terms of the transition rates *q*_*ij*_ given above in Equation (2).

### 2.3. Direct calculation of conditional activity from the transition rates

Given the transition rates *q*_*ij*_, for each binding site *a* define the transition rate matrix *Q*^*a*^ where transitions that change the binding configuration of site *a* are disallowed:

$${\left(Q^{a}\right)}_{ij}=\{\begin{array}{ll} q_{ij} & i\neq j\;\text{and microstates }i,j\text{ have site }a\text{ in}\\ & \text{ the same binding configuration}\\ 0 & i\neq j\;\text{and microstates }i,j\text{ have site }a\text{ in}\\ & \text{ different binding configurations}\\ -\sum_{k\neq i}q_{ik} & i=j \end{array}$$

The state space of all 1200 microstates may partitioned into subspaces, where each subspace is a connected component of the transition graph where transitions that change the binding configuration of site *a* are disallowed. The *Q*^*a*^ matrices are block diagonal (under a relabeling of the indices), and each block represents the transitions within a given subspace. In the language of Markov chain theory, each of these blocks represents a Markov chain where the transitions that change the binding configuration of site *a* have been made absorbing. Using techniques for analyzing absorbing Markov chains (Kemeny and Snell, 1961; Tavare, 1979; Syski, 1992), we may express the exchange and persistence times in terms of the fundamental matrix for a regular Markov chain *N*^*a*^ = −(*Q*^*a*^)^−1^.

We now define

(7) $$B^{a}=N^{a}R^{a}$$

(8) $$K_{i}^{a}=\frac{\sum_{j}K_{j}{\left(R^{a}\right)}_{ji}}{\sum_{j}\sum_{k}K_{j}{\left(R^{a}\right)}_{jk}}$$

where the matrices *R*^*a*^ encode the transitions between subspaces:

$${\left(R^{a}\right)}_{ij}=\{\begin{array}{ll} q_{ij} & \begin{array}{l} i\neq j\;\text{and microstates }i,j\text{ have site }a\text{ in different}\\ \text{ binding configurations} \end{array}\\ 0 & \text{otherwise.} \end{array}$$

(*B*^*a*^)_*ij*_ is the probability that *j* is the first microstate in which the binding configuration of site *a* has changed, given that the system has started in microstate *i*. *K*^*a*^_*i*_ represents the probability of finding the system in microstate *i* just after the binding configuration of *a* has changed.

We may now define the persistence time

(9) $$\tau_{p}[a]=\frac{1}{\tau^{a}}\sum_{i}\sum_{j}\sum_{k}K_{i}^{a}{\left(N^{a}\right)}_{ij}{\left(N^{a}\right)}_{jk}$$

and exchange time

(10) $$\tau_{x}[a\leftarrow b]=\frac{1}{\tau^{b}}\sum_{i}\sum_{j}\sum_{k}\sum_{l}K_{i}^{b}{\left(N^{b}\right)}_{ij}{\left(B^{b}\right)}_{jk}K_{k}^{a}{\left(N^{a}\right)}_{kl}$$

where τ^*a*^ = ∑_*i*_ ∑_*j*_ *K*^*a*^_*i*_(*N*^*a*^)_*ij*_ is a normalization. The conditional activity then follows from Equation (6).

(See the Supplemental Material for a derivation).

### 2.4. Simulation

The conditional activity and mutual information may be directly calculated from the Markov model, as described above, or from simulated or experimental time-series data. For a reduced five binding site model, the analytic calculations presented above agreed with the same quantities calculated from simulated time-series data (Supplementary Figure 3) simulated using the Gillespie algorithm (Gillespie, 1977).

## 3. Results

The lysogenic and lytic pathways exhibit differing levels of fluctuations, as can be seen qualitatively in Figure 2. In the lysogenic pathway, the system is stabilized by long-range DNA looping—cooperativity between the left and right operators—and cooperative interactions between adjacent sites on each operator (Santillán and Mackey, 2004; Anderson and Yang, 2008; Zurla et al., 2009; Norregaard et al., 2013). With more than 99% probability the system exists in one of three microstates exhibiting an octomeric configuration of CI bound to *O*_R1_, *O*_R2_, *O*_L1_, and *O*_L2_ (Supplementary Figure 1).

**Figure 3:**
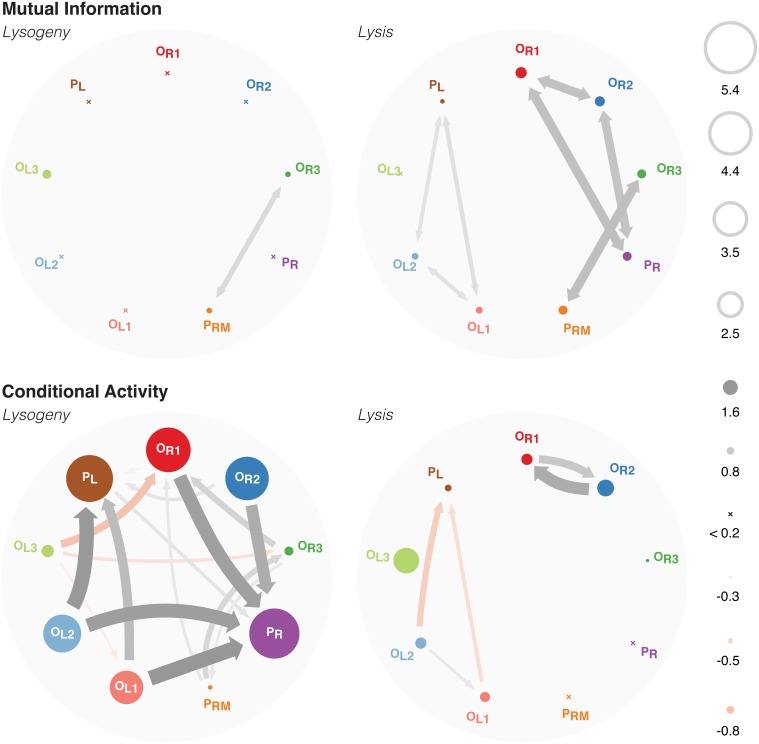
**Top**: A graph showing the mutual information between each binding site. The entropies of each binding site is shown as the size of the circle. **Bottom:** A graph of the conditional activity between each binding site. *CA*[*a* ← *b*] is drawn as an arrow from *b* to *a*, whereas *CA*[*a* ← *a*] is shown as the size of *a*. See Supplementary Figure 2 for the same data shown in matrix form.

This high degree of stability is quantified by the entropies of each binding site (Figure 3 top left). Most binding sites are fixed in one microstate with high probability. Only the *P*_RM_, *O*_R3_, and *O*_L3_ sites are fluctuating, as shown by their non-negligible entropy. The high mutual information between *P*_RM_ and *O*_R3_ represents the near perfect anticorrelation between the two sites: one is almost always bound and no more than one may be bound at a time because they physically block each other.

Similarly, in the lytic pathway, the mutual information (Figure 3 top right) clearly indicates that *P*_R_ overlaps with, and hence is anticorrelated with, both *O*_R1_ and *O*_R2_; *P*_RM_ overlaps with *O*_R3_; and *P*_L_ overlaps with *O*_L1_ and *O*_L2_. The mutual information between *O*_R1_ and *O*_R2_, and that between *O*_L1_ and *O*_L2_, arise because when those sites are unbound by their overlapping RNAP, the high concentration of Cro leads to both sites being bound simultaneously with Cro. Note that this simultaneous binding is highly correlated, but does not represent any cooperative binding effects: there is cooperativity between binding at *O*_R1_ and *O*_R2_, and between *O*_R2_ and *O*_R3_, yet there is only mutual information between the former pair, that which overlaps *P*_R_. Similarly, there is cooperativity between *O*_L1_ and *O*_L2_, and between *O*_L2_ and *O*_L3_, and again we only see mutual information between the former pair, that which overlaps *P*_L_. This indicates that correlations indicated by high mutual information are due solely to high concentrations of Cro, leading to simultaneous binding as soon as the blocking RNAP unbinds.

We see that the mutual information is sensitive only to correlations that occur between physically-overlapping binding sites. Since these overlaps were explicitly introduced when constructing the model, the mutual information does not give any new information. The conditional activity, however, shows a rich network of interactions between binding sites that is not evident directly from the model. Furthermore, unlike the mutual information, the conditional activity is highly informative even when the system is in a highly stable regime (as is the lysogenic pathway), because it is sensitive to fluctuations away from equilibrium behavior even when they are rare.

In the lysogenic pathway (Figure 3 lower left), the conditional activity maps out the interactions which can lead the system to switch to lysis. Lysogeny is characterized by the maintenence of a stable level of CI by RNAP binding to *P*_RM_, and strong repression of Cro production. Thus, RNAP is bound to *P*_RM_ with 86% probability, whereas RNAP is bound to *P*_R_ with probability ≤10^−5^. Because RNAP binding to *P*_R_ leads to production of Cro, and high levels of Cro lead to lysis, those events represent fluctuations of the system toward the lysis pathway.

*P*_R_ physically overlaps with *O*_R1_ and *O*_R2_, so both must be unbound before RNAP may bind to *P*_R_. However, *O*_R1_ and *O*_R2_ are kept bound with CI by cooperative binding with each other and with *O*_L1_, *O*_L2_. An unbinding event of *O*_R1_, *O*_R2_, *O*_L1_, or *O*_L2_ therefore represents a deviation from this highly-stable octomeric configuration, incurring the associated penalty of breaking multiple cooperative binding interactions. Once the octomer is disrupted, it is more likely that *O*_R1_ and *O*_R2_ will unbind, thus allowing RNAP to bind to *P*_R_. This is indicated by the large CA arrows originating at *O*_R1_, *O*_R2_, *O*_L1_, and *O*_L2_ and pointing at *P*_R_.

The binding behavior of *P*_R_ is highly non-Markovian, as quantified by *CA*[*P*_R_ ← *P*_R_] ≈ 5.4, indicating that the binding state of *P*_R_ changes rarely, but just after it changes it will change again ~ 10^5^ times sooner than it would have otherwise. This large conditional activity is due to a long persistence time. Since RNAP binding at *P*_R_ and the resulting production of Cro serves to move the system from lysogeny to lysis, this large persistence time provides a measure of the stability of the lysogenic pathway. The much shorter exchange time indicates that RNAP binding at *P*_R_ is controlled by other cooperative binding processes, which are revealed by the off-diagonal elements of the CA matrix (see below).

Similarly, *O*_L1_ and *O*_L2_ have CA arrows pointing at *P*_L_ since they physically overlap *P*_L_, and hence their unbinding makes RNAP more likely to bind at *P*_L_. The octomeric configuration with *O*_R3_ either bound by CI or unbound is the dominant behavior in the lysogenic pathway. *O*_L3_ is slightly more likely to be bound with CI than unbound, whereas is *O*_R3_ significantly more likely unbound than bound with CI. Thus, when the binding configuration of *O*_L3_ changes, it is more likely to be an unbinding event, which eliminates the cooperativity between CI at *O*_L3_ and *O*_R3_ which could recruit CI to bind to *O*_R3_. Hence, we see a negative CA arrow from *O*_L3_ to *O*_R3_.

The conditional activity in the lysis pathway (Figure 3 bottom right) shows a much smaller degree of non-Markovian behavior than that seen in the lysogenic pathway. The small, positive CA arrows between *O*_R1_ and *O*_R2_, and between *O*_L1_ and *O*_L2_, represent the same correlated binding shown by the mutual information. The small, negative CA originating at *O*_L1_ and *O*_L2_ and pointing at *P*_L_ represent the same correlations due to overlap as were detected by the mutual information. (There are also small, negative CAs originating at *P*_R_ and pointing at *O*_R1_ and *O*_R2_, too small to be plotted in this figure. They are visible in Supplementary Figure 2). The conditional activity is evidently diminished for systems in which fluctuations are common; this is the regime correspond to large entropies and mutual information values.

The mutual information and conditional activity thus provide orthogonal, and complimentary, representations of the behavior of the system. Because cooperativity should lead to correlated binding, we expected the mutual information to recover cooperative interactions between binding sites. However, we find that mutual information fails to do so. Cooperativity does lead to correlations between binding activity at different sites. However, cooperativity is also a strongly stabilizing effect, leading to the system residing in one of a few microstates which maximize cooperative interactions, and hence to small entropies. Because the mutual information between two binding sites is bounded above by the entropy of each binding site, this implies that systems exhibiting strong cooperative interactions are also likely to show little mutual information. By looking for correlations in the binding times instead of correlations between equilibrium binding states, the conditional activity is sensitive to the rare fluctuations which reveal the effects of cooperativity.

## 4. Discussion

We designed the conditional activity to be a quantitative measure of the deviation from Markovian dynamics. While the Markov model of transitions between the 1200 microstates is Markovian by construction, the cooperative binding and overlap of binding sites encoded in the transition rate matrix lead to highly non-Markovian behavior when the space of 1200 microstates is projected down to the space of the nine binding sites. Because non-Markovian dynamics are a general feature of systems exhibiting cooperativity, the conditional activity can be used to study the interactions and flow of information in such systems. Furthermore, since the conditional activity is an efficiently-computable low-dimensional representation of a high-dimensional stochastic model that preserves important information about the dynamical behavior of the system, it is an ideal tool for model inference and model reduction of biochemical systems. Since simulating the Markov model is computationally expensive or intractable for large models with widely-separated time scales, a major contribution of this paper is deriving an analytic expression for the conditional activity in terms of the Markov transition matrix for the microstates of the system, bypassing the need for simulations.

Cooperativity in biochemical systems often results in highly stable configurations of macromolecular complexes. In this work, we showed that rare fluctuations away from these stable configurations contain information about important interactions between binding sites and mechanisms of switching between lysogeny and lysis in phage lambda. We find that the conditional activity is sensitive to these fluctuations, whereas the mutual information is not. Our results illustrate the limited utility of the mutual information in characterizing systems with strong cooperative interactions. In Lin (under review), we showed that proteins exhibit non-Markovian dynamics on the intramolecular scale, and that the conditional activity effectively characterized the flow of information through these molecular systems. In this work, we find cooperativity leads to non-Markovian dynamics at the intermolecular level, and motivate the conditional activity as a tool for studying information flow through gene regulatory networks.

For the model of phage lambda, the energies of each of the 1200 microstates were known experimentally, allowing for the construction of a full Markov model involving the rates of transition between microstates. Therefore, we could calculate the correlation functions between the different binding sites directly from the transition rate matrix as well as from explicitly simulating the Markov model. By recording the times of binding events for pairs of binding sites, the conditional activity may be measured experimentally. Since cooperativity and the resulting stability are typical features of biochemical systems, the limitations of mutual information we encounter here will apply equally to those systems, and we expect the methods we present here to be useful in analyzing a wide variety of such systems.

The *lac* operon in *E. coli* is a bistable switch with an almost identical architecture to that of the phage lambda switch we have considered here (except its most stable configuration is a DNA loop bound by a repressor tetramer; it lacks the octomeric configuration of the phage lambda switch). Just as our analysis of the phage lambda switch found that Cro production resulted from unbinding of CI repressor from the highly-stable octomer in the looped-DNA configuration, Choi et al. (2008) reach the same conclusion experimentally in the *lac* system. Their single-molecule experiment observed bursts of protein production following unbinding of the repressor from the tetrameric configuration.

Our results demonstrate that one may infer such behavior without directly observing it experimentally, as it is revealed in temporally-correlated binding activity when the system is fluctuating around a stable configuration—the conditional activity elucidates the architecture of the bistable switch without actually observing it switch. Furthermore, our analysis suggest that in some cases it may not be necessary to perform a difficult single-molecule time-series experiment to directly measure the conditional activity in gene regulatory networks. All our results were calculated using thermodynamic parameters alone (binding energies), which in some cases may be obtained *in vitro* and are often easier to measure than real-time single-molecule binding activity. We suspect that the strategy of inferring kinetics from thermodynamic parameters by assuming detailed balance and equal binding rates, and then calculating dynamic quantities from these kinetics, is valid for a large class of biochemical systems. It remains for future work to explore the limits of this approximation (see e.g., Daniels et al., 2008).

Colquhoun and Hawkes (1981) and subsequent work on ion channels compellingly demonstrated how non-Markovian behavior could bridge scales in biological systems: by measuring non-Markovian dynamics on observable scales one could place strong constraints on microscopic behavior which may not be directly accessible experimentally. This line of work leverages explicit stochastic models of ion channels to relate observable dynamics with microscopic parameters, so that the latter may be inferred from the former. The conditional activity, however, was designed to detect interactions between parts of the system in a model-independent way.

Because the *lac* system is well-studied and relatively simple, Choi and coauthors were able to hypothesize that the dissociation of the repressor tetramer would lead to bursts of protein production, and could design an experiment to observe this phenomenon. As one considers more complex and less well-understood biochemical networks, it becomes highly non-intuitive to design an appropriate experiment to observe the desired phenomena, since the interactions between the components in the network may not be known. By identifying the functional connectivity between the elements in a biochemical network, the conditional activity could serve to guide the design of such experiments. Developmental gene regulatory networks are a particularly interesting application of this approach. For the gene network which controls cell-fate determination in *Arabidopsis*, the conditional activity would suggest the perturbations to which the system is insensitive and also those experimental interventions which would most efficiently lead to specific cell fates.

In future work, we also aim to move beyond systems with established models and seek to demonstrate the efficacy of the conditional activity in characterizing systems for which a full Markov model is not yet known. This approach has promise in neural systems, where almost all of the information in the network is encoded by the times of spike events and effective experimental protocols exist for recording spike times for large networks of neurons. Previous work has shown how correlations between subsequent waiting times (Farkhooi et al., 2009; Schwalger and Lindner, 2010) reveal the dynamics of a single neuron; our results suggest that correlations of waiting times between different neurons may reveal the functional connectivity of the network.

### Conflict of interest statement

The authors declare that the research was conducted in the absence of any commercial or financial relationships that could be construed as a potential conflict of interest.
