# Supplementary material for: Cooperativity leads to temporally-correlated fluctuations in the bacteriophage lambda genetic switch
Source: Front Plant Sci. 2015 Apr 8;6:214. doi: 10.3389/fpls.2015.00214 (PMC4389348; doi:10.3389/fpls.2015.00214)
Supplement: Supplementary file 1 [file DataSheet1.PDF]

Frontiers in Plant Science

Research Article
9 March 2015

Supplementary Material: Cooperativity Leads to Temporally-Correlated Fluctuations in the Bacteriophage Lambda Genetic Switch

Jacob Quinn Shenker[1] **and Milo M. Lin**[2,*]

[1] *Department of Physics, California Institute of Technology, Pasadena, CA, USA*
[2] *Pitzer Center for Theoretical Chemistry, University of California, Berkeley, CA, USA*

Correspondence*:
Milo M. Lin
Pitzer Center for Theoretical Chemistry, 39 Gilman Hall, University of California, Berkeley, CA, 94720, USA, milolin@berkeley.edu

1 MODEL PARAMETERS

The concentrations of CI, Cro, and RNAP that correspond to the lysogenic and lytic pathways are obtained using the model and parameters from **Santillán and Mackey** (2004). The concentrations of CI, Cro, and RNAP that correspond to the lysogenic and lytic pathways are found by solving $\Phi([CI_T],[Cro_T]) = \Theta([CI_T],[Cro_T]) = 0$ where

$$\Phi([CI_T],[Cro_T]) = \frac{\nu_{\mathrm{cI}}}{\gamma_{\mathrm{M}}+\mu}[O_{\mathrm{R}}](k^{\mathrm{q}}_{\mathrm{cI}} f^{\mathrm{q}}_{\mathrm{RM}} + k^{\mathrm{s}}_{\mathrm{cI}} f^{\mathrm{s}}_{\mathrm{RM}}) - (\gamma_{\mathrm{cI}}+\mu)[CI]_T \tag{1}$$

$$\Theta([CI_T],[Cro_T]) = \frac{\nu_{\mathrm{cro}}}{\gamma_{\mathrm{M}}+\mu}[O_{\mathrm{R}}]k_{\mathrm{cro}} f_{\mathrm{R}} - (\gamma_{\mathrm{cro}}+\mu)[Cro]_T \tag{2}$$

and $[CI_T]$, $[Cro_T]$ are the total concentrations of CI and Cro monomers (including those in dimers), with

$$[CI_2] = \frac{1}{2}[CI] - \frac{K^{\mathrm{cI}}_{\mathrm{D}}}{8}\left(\sqrt{1+8\frac{[CI_T]}{K^{\mathrm{cI}}_{\mathrm{D}}}}\right) \tag{3}$$

$$[Cro_2] = \frac{1}{2}[Cro] - \frac{K^{\mathrm{cro}}_{\mathrm{D}}}{8}\left(\sqrt{1+8\frac{[Cro_T]}{K^{\mathrm{cro}}_{\mathrm{D}}}}\right). \tag{4}$$

All parameters are defined and given in **Santillán and Mackey** (2004), choosing the repressor degradation rate $\gamma_{\mathrm{cI}} = 0.015\ \mathrm{min}^{-1}$, which lies in the range consistent with bistability (see also **Gedeon et al.** (2008), which adopts the same model). The lysogenic pathway is identified as the fixed point with high CI and low Cro; the lytic pathways is identified as the fixed point with low CI and high Cro. We find that lysogenic conditions are described by $[CI_2] \simeq 0.16\ \mu\mathrm{M}$ and $[Cro_2] \simeq 9\times10^{-11}\ \mu\mathrm{M}$ while lytic conditions are described by $[CI_2] \simeq 3\times10^{-3}\ \mu\mathrm{M}$ and $[Cro_2] \simeq 0.2\ \mu\mathrm{M}$. We validated our implementation of this model against the results and figures given in **Santillán and Mackey** (2004) and **Gedeon et al.** (2008).

2 DERIVATION OF THE DIRECT EXPRESSION FOR CONDITIONAL ACTIVITY (EQUATIONS 9 AND 10)

In the following, for clarity we write matrix multiplication explicity as sums. We use Q^a, R^a, N^a, B^a, and K_i^a as defined in the main text. The Q^a represent the transition rate matrices of a Markov chain where transitions that change the binding configuration of site a are made absorbing. Given that the chain begins in the microstate i, the time until absorption has mean $\tau_i^a \equiv \sum_j (N^a)_{ij}$ and variance $\nu_i^a \equiv 2\sum_j \sum_k (N^a)_{ij}(N^a)_{jk}$.

$$\tau_p[a] \equiv \frac{1}{2\tau}\sum_{t=1}^{N(a)} W(a, T(a,t))^2 = \frac{\frac{1}{N(a)}\sum_{t=1}^{N(a)} W(a, T(a,t))^2}{2\tau/N(a)}$$

Rewritten this way, it is clear that the persistence time is simply the mean squared waiting time between transitions that change the binding configuration of site a normalized by the twice the mean waiting time (recall that τ is the total observation time). At each transition time $T(a,t)$ for each index $1 \leq t \leq N(a)$, we know only that the binding configuration of site a has just changed. Since the system is in thermodynamic equilibrium, the probability that the system is in microstate i at any transition time $T(a,t)$ is simply the fraction of flux along transitions that change the configuration of a incoming into microstate i:

$$K_i^a = \frac{\sum_j K_j (R^a)_{ji}}{\sum_j \sum_k K_j (R^a)_{jk}}$$

Thus as $N(a) \to \infty$,

$$\frac{1}{N(a)}\sum_{t=1}^{N(a)} W(a, T(a,t))^2 \to \sum_i K_i^a \nu_i^a$$

$$\tau/N(a) \to \sum_i K_i^a \tau_i^a$$

We conclude

$$\tau_p[a] = \frac{\sum_i \sum_j \sum_k K_i^a (N^a)_{ij} (N^a)_{jk}}{\sum_i \sum_j K_i^a (N^a)_{ij}}. \tag{5}$$

Now we consider the exchange time:

$$\tau_x[a \leftarrow b] = \frac{1}{\tau}\sum_{t=1}^{N(b)-1} W(a, T(b, t+1)) W(b, T(b,t)). \tag{6}$$

Take $N(a) \to \infty$ and fix arbitrary t. The expression for the exchange time involves three times: T_a, the initial transition time of b; T_b', the first subsequent transition time of b; and T_a, the first transition time of a after $T(b, t+1)$. Now the t-th term of the sum in Equation 6 is $W(a, T(b, t+1)) W(b, T(b,t)) = (T_a - T_b')(T_b' - T_b)$.

At T_b, the configuration of b has just changed, so the system is in microstate i with probability K_i^b. Given that the system begins in microstate j, the mean time until changing the configuration of b again $(T_b' - T_b)$

is τ_j^b. Upon this second change of b at time T_b', probability of the system residing in microstate k is $(B^b)_{jk}$. Given that the system is in microstate k at T_b', the mean time until the configuration of a changes is τ_k^a.

Averaging over all microstates j and k,

$$\big\langle (T_a - T_b')(T_b' - T_b) \big\rangle = \sum_j \sum_k (K_j^b (B^b)_{jk} K_k^a)(\tau_j^b \tau_k^a)$$

where on the right-hand side the first factor in parentheses is the probability that the system will visit microstates j and k at times T_b and T_b', respectively, and the second is the product of waiting times given that the system visits those microstates.

Writing in terms of the fundamental matrices N^a, we have

$$\tau_x[a \leftarrow b] = \frac{\sum_i \sum_j \sum_k \sum_l K_i^b (N^b)_{ij} (B^b)_{jk} K_k^a (N^a)_{kl}}{\sum_i \sum_j K_i^b (N^a)_{ij}}. \quad (7)$$

See (**Syski**, 1992; **Kemeny and Snell**, 1961; **Tavare**, 1979) for techniques for calculating sojourn times for continuous-time Markov chains. **Colquhoun and Hawkes** (1981); **Ball et al.** (2000) applies these techniques to compute similar quantities in the context of models for ion channels in cell membranes.

We validate the direct expression for conditional activity (equations 9 and 10 in the main text) against conditional activities calculated from simulation. The nine binding site model for phage lambda is computationally expensive to simulate long enough to calculate accurate conditional activities. As such, we do this comparison for the five binding site model (**Tian and Burrage**, 2004). We find that the simulation-based CA converges to the direct CA calculation (Supplementary Figure 3).

REFERENCES

Ball, F. G., Milne, R. K., and Yeo, G. F. (2000), Stochastic models for systems of interacting ion channels, *Mathematical Medicine and Biology*, 17, 3, 263–293

Colquhoun, D. and Hawkes, A. G. (1981), On the stochastic properties of single ion channels, *Proceedings of the Royal Society of London. Series B. Biological Sciences*, 211, 1183, 205–235, doi:10.1098/rspb.1981.0003

Gedeon, T., Mischaikow, K., Patterson, K., and Traldi, E. (2008), Binding Cooperativity in Phage λ is Not Sufficient to Produce an Effective Switch, *Biophysical Journal*, 94, 9, 3384–3392

Kemeny, J. G. and Snell, J. L. (1961), Finite continuous time Markov chains, *Theory of Probability & Its Applications*, 6, 1, 101–105

Santillán, M. and Mackey, M. C. (2004), Why the Lysogenic State of Phage λ Is So Stable: A Mathematical Modeling Approach, *Biophysical Journal*, 86, 1, 75–84

Syski, R. (1992), Passage Times for Markov Chains (IOS Press)

Tavare, S. (1979), A note on finite homogeneous continuous-time Markov chains, *Biometrics*, 831–834

Tian, T. and Burrage, K. (2004), Bistability and switching in the lysis/lysogeny genetic regulatory network of bacteriophage λ, *Journal of Theoretical Biology*, 227, 2, 229–237

3 SUPPLEMENTARY TABLES AND FIGURES

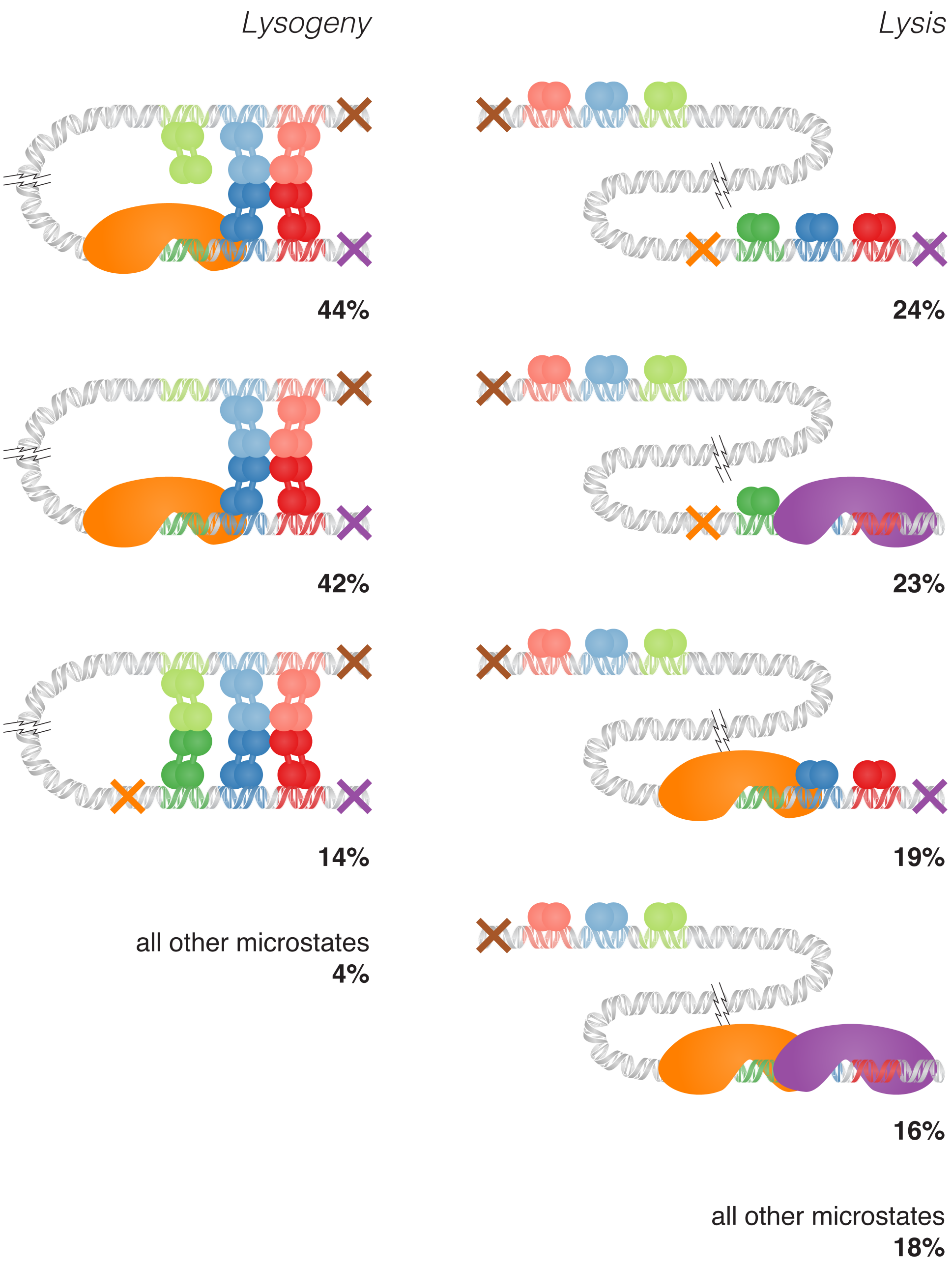

Supplementary Figure 1. Microstates with probabilities in excess of 4% under lysogenic (left) and lytic (right) conditions.

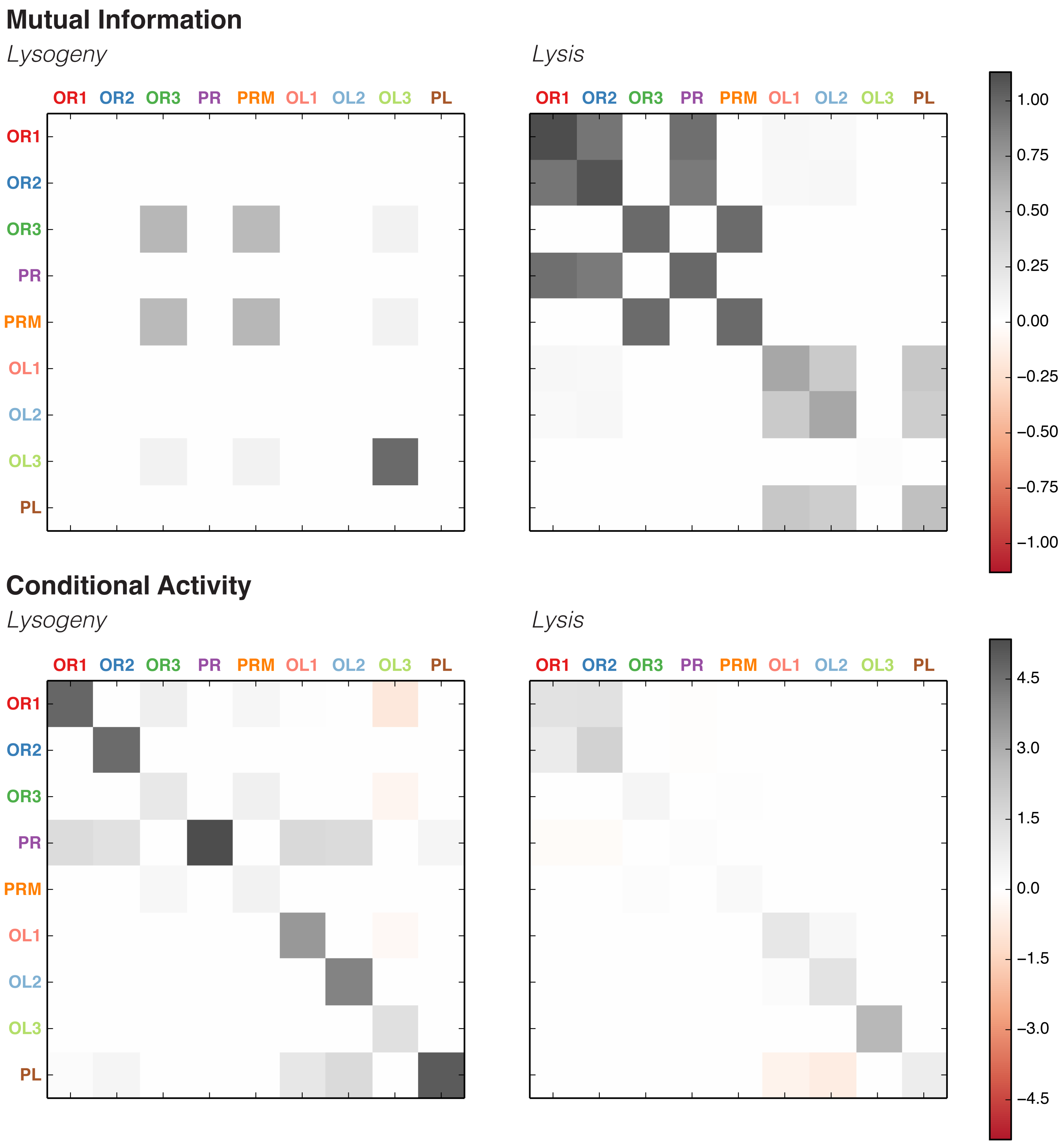

Supplementary Figure 2. The same as Figure 3, shown in matrix form. For conditional activity, the matrix is oriented such that each matrix element represents the conditional activity of the row degree-of-freedom conditional on the column degree-of-freedom (i.e., $CA[a \leftarrow b]$ lies in row a, column b).

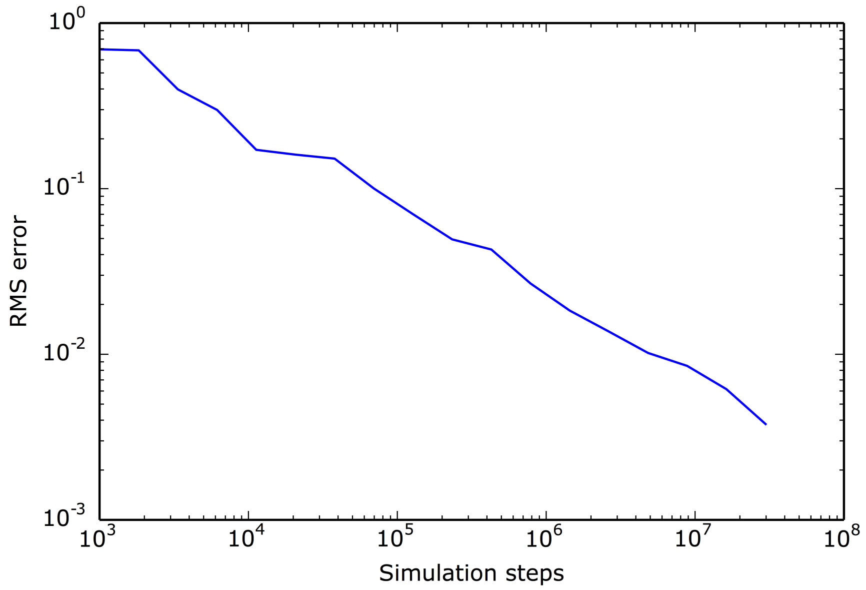

Supplementary Figure 3. The RMS error of conditional activity calculated from simulations. As more timesteps are simulated, the CA obtained from simulation converges with the analytic expression for CA.
